# Mammal virus diversity estimates are unstable due to accelerating discovery effort

**DOI:** 10.1098/rsbl.2021.0427

**Published:** 2022-01-05

**Authors:** Rory Gibb, Gregory F. Albery, Nardus Mollentze, Evan A. Eskew, Liam Brierley, Sadie J. Ryan, Stephanie N. Seifert, Colin J. Carlson

**Affiliations:** ^1^ Centre on Climate Change and Planetary Health, London School of Hygiene and Tropical Medicine, London, UK; ^2^ Centre for Mathematical Modelling of Infectious Diseases, London School of Hygiene and Tropical Medicine, London, UK; ^3^ Department of Biology, Georgetown University, Washington, DC; ^4^ Medical Research Council - University of Glasgow Centre for Virus Research, Glasgow, UK; ^5^ Institute of Biodiversity, Animal Health and Comparative Medicine, College of Medical, Veterinary and Life Sciences, University of Glasgow, Glasgow, UK; ^6^ Department of Biology, Pacific Lutheran University, Tacoma, WA, USA; ^7^ Department of Health Data Science, University of Liverpool, Liverpool, UK; ^8^ Department of Geography, University of Florida, Gainesville, FL, USA; ^9^ Emerging Pathogens Institute, University of Florida, Gainesville, FL, USA; ^10^ College of Life Sciences, University of KwaZulu Natal, Durban 4041, South Africa; ^11^ Paul G. Allen School for Global Health, Washington State University, Pullman, WA, USA; ^12^ Center for Global Health Science and Security, Georgetown University Medical Center, Washington, DC, USA; ^13^ Department of Microbiology and Immunology, Georgetown University Medical Center, Washington, DC, USA

**Keywords:** host-virus association, mammals, virus, zoonotic, chiroptera, discovery effort

## Abstract

Host-virus association data underpin research into the distribution and eco-evolutionary correlates of viral diversity and zoonotic risk across host species. However, current knowledge of the wildlife virome is inherently constrained by historical discovery effort, and there are concerns that the reliability of ecological inference from host-virus data may be undermined by taxonomic and geographical sampling biases. Here, we evaluate whether current estimates of host-level viral diversity in wild mammals are stable enough to be considered biologically meaningful, by analysing a comprehensive dataset of discovery dates of 6571 unique mammal host-virus associations between 1930 and 2018. We show that virus discovery rates in mammal hosts are either constant or accelerating, with little evidence of declines towards viral richness asymptotes, even in highly sampled hosts. Consequently, inference of relative viral richness across host species has been unstable over time, particularly in bats, where intensified surveillance since the early 2000s caused a rapid rearrangement of species' ranked viral richness. Our results illustrate that comparative inference of host-level virus diversity across mammals is highly sensitive to even short-term changes in sampling effort. We advise caution to avoid overinterpreting patterns in current data, since it is feasible that an analysis conducted today could draw quite different conclusions than one conducted only a decade ago.

## 1. Introduction

Pathogens are unevenly distributed across host species, and understanding the underlying coevolutionary processes is important for both ecological and health-motivated research. For example, data on how viral diversity is distributed across species and geographies can provide insights into biogeographical trends and anthropogenic drivers of cross-species transmission and disease emergence [1–3]. Researchers have developed numerous hypotheses about the mechanisms underlying observed differences in virus diversity across hosts, from broad macroevolutionary trends (e.g. bats host a greater apparent diversity of viruses than other mammal orders [4]) to narrower ecological associations (e.g. longer lived bats living in larger groups host a greater apparent diversity of viruses [5]). Such work frequently analyses the number of viruses known to infect a given host species (viral richness) by synthesizing existing host-virus association data [1–7].

However, recent work has raised concerns that such datasets inspire false confidence. Although host-virus association datasets take an increasingly complete inventory of current scientific knowledge [8], a substantial proportion of known viruses remain excluded because of long lead times before official taxonomic recognition, which is itself not uniform across the virome [9]. An even greater proportion of the global virome remains completely undescribed [10,11], with current knowledge strongly influenced by discovery strategies [12]. This may undermine inference about the distribution of zoonotic risk among host taxa [9], and multiple studies have shown that apparent patterns in zoonotic virus richness become insignificant after adjusting for total viral richness [13,14]. Yet it remains unclear how this impacts more basic scientific questions, including those concerning macroecological patterns in species-level viral diversity.

In this study, we evaluate whether—given the limits of current data—host-level estimates of viral diversity in mammals can be considered biologically meaningful based on their temporal consistency. Even when a species' total viral diversity has been ground-truthed by thorough metagenomic sampling and rarefaction-based estimation [15], estimates suggest only approximately 3–7% of their viruses are captured by current host-virus association data [10]. With such a small proportion of viruses described, it is plausible that comparative studies of viral diversity are using numbers that are both subject to change and highly sensitive to differences in sampling strategies between different host and virus groups.

We explore these questions using a dataset of 6571 mammal host-virus associations and their year of discovery (the earliest year that a virus was reported in association with a given host), representing a comprehensive inventory of known associations from 1930 to 2018 (electronic supplementary material, figure S1). We focus on wild mammals, because the historical intensity of pathogen discovery effort on domestic species could confound inference (electronic supplementary material, figure S2). First, we examine virus accumulation curves to test whether current absolute viral richness estimates in well-sampled orders and species are likely to be accurate, applying a test borrowed from research on parasite biodiversity [16,17]: richness estimates can only be taken as ‘stable’—and thus reflective of values close to the truth—if accumulation curves have passed an inflection point towards an asymptote [18]. Alternatively, if viral diversity is still accumulating exponentially, current estimates may have little correlation to ‘true’ (unknown) viral richness. Second, we evaluate the temporal stability of relative viral richness estimates across wild mammals by testing the rank correlation between present-day and historical estimates. If the correlation of relative viral richness has remained fairly stable over time, this would suggest that species' viromes have been sampled proportionally, and that current data can (despite being incomplete) still provide meaningful comparative information about viral diversity across mammals.

## 2. Methods

### (a) Mammal host-virus association data over time

We accessed mammal host-virus records (1277 mammal species and 1756 viruses, of which 1073 are currently ratified by the International Committee on the Taxonomy of Viruses, ICTV) from a comprehensive multi-source database of host-virus associations (VIRION; https://github.com/viralemergence/virion). VIRION compiles data from several static data sources, the NCBI GenBank database and the USAID PREDICT project database, with host taxonomy standardized to the NCBI taxonomic backbone [6,8]. Here, we define a host-virus association based on broad evidence of infection: either serological, polymerase chain reaction (PCR)-based, or viral isolation. Some records describe recently discovered viral strains that are not yet resolved to species level; to ensure these do not inflate viral richness estimates, we only included taxonomically resolved viruses, defined as either ratified by ICTV (*n* = 1073) or reconciled to the internal viral taxonomy of the PREDICT project (*n* = 683) [8].

We defined the ‘discovery year’ for each unique host-virus pair (*n* = 6571) as the earliest year a given virus was reported in a given host, based on date of publication (for literature-based records), accession (for NCBI Nucleotide and GenBank-based records), or sample collection (for records from the USAID PREDICT database). The full database contains data up to mid-2021; however, novel association records become notably sparser after 2018 (electronic supplementary material, figure S1), probably owing to delays between viral sampling, reporting and taxonomic assignment [6]. We therefore excluded all post-2018 records to avoid biasing inference about virus discovery trends in recent years. To examine trends in publication effort (a proxy for sampling effort), for each host we extracted annual counts of virus-related publications (by searching for species binomial plus all synonyms *and ‘*virus' or ‘viral’) from the PubMed database using the R package ‘rentrez’ [19]. We visualized cumulative virus discovery curves and publication counts over time at order-level (electronic supplementary material, figures S2 and S3) and across all wild mammal species (electronic supplementary material, figures S4 and S5). With the exception of individual species-level models (electronic supplementary material, figure S6), all subsequent analyses included wild species only (*n* = 1246) and excluded domestic and common laboratory species (defined using metadata compiled for VIRION [6]) (electronic supplementary material, figure S2).

### (b) Modelling trends in viral discovery rates at order- and species-level

**Figure 1.**
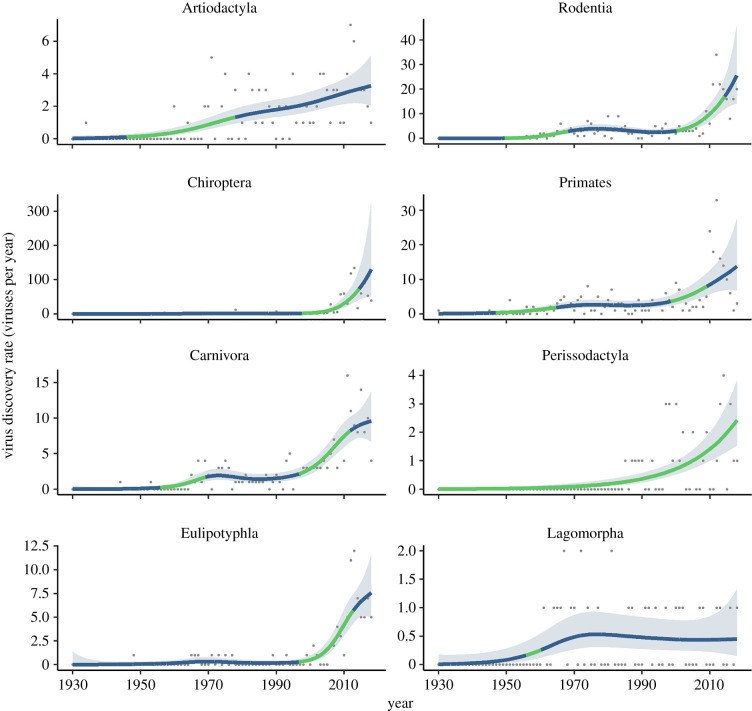
Virus discovery rates within well-sampled mammal orders are still either constant or accelerating. Points show the number of novel viruses discovered per year (1930–2018) infecting wild species of each of the eight most virus-rich mammalian orders. Lines and shading show the fitted trend in virus discovery rate (mean and pointwise 95% confidence interval; see Methods). Line colour indicates periods with strong evidence of either an upwards (green) trend in discovery rates (95% confidence interval of the first derivative of the fitted trend above zero) or no significant trend (blue). Note the different *y*-axis scales for each graph.

We modelled trends in viral discovery rates by fitting generalized additive models (GAMs) to annual counts of viruses discovered per taxon (1930–2018), with a nonlinear trend of year fitted using penalized thin-plate regression splines in ‘mgcv’ [20]. We fitted models at order-level (including the top eight best-sampled mammal orders with the highest known viral richness: Artiodactyla, Rodentia, Carnivora, Primates, Chiroptera, Lagomorpha, Perissodactyla and Eulipotyphla), and at species-level for the top 50 most virus-rich species in our dataset. Virus discovery counts were modelled as a Poisson process for all orders except Chiroptera, Rodentia and Primates, which were modelled using a negative binomial likelihood due to high overdispersion in recent years (figure 1). If discovery curves have reached an inflection point in any taxon, we would expect a consistent downward trend in discovery rates in recent years. To test this, we identified time periods showing strong evidence of either increasing or declining trends, defined as periods during which the 95% confidence interval of the first derivative of the fitted spline does not overlap zero.

### (c) Evaluating the temporal stability of relative viral richness estimates across taxa

A key assumption of most ecological studies using host-virus data is that currently known differences in virome composition between species (or higher groupings) are broadly representative of ‘true’ underlying patterns in viral diversity. If this were the case, differences in relative viral richness across taxa would be expected to stay relatively stable over time, even as discovery effort fills the gaps in species-level virus inventories. Alternatively, uneven sampling effort across species and time may severely impact this assumption [9], by causing instability and rapid reordering of viral richness estimates across taxa. We tested this by calculating the rank correlation (Spearman's *ρ*) of viral richness in 2018 to viral richness estimates in annual timesteps backwards to 1960 (i.e. comparing the similarity of each annual historical ‘snapshot’ of ranked viral richness to present-day knowledge). We conducted this analysis at several taxonomic levels, comparing viral richness at the species level (across all mammal species, and separately within each of the key orders listed above), and comparing two different metrics at family and order levels (total viral richness and mean species-level viral richness).

### (d) Examining the stability of ecological inferences

As a test of how changing knowledge might impact ecological inference, we examined the relationship between order-level species richness and viral richness, using data summarized at 5-year increments between 1990 and 2020 (*n* = 17 orders). We aimed to replicate Mollentze & Streicker's [13] finding that, at order-level, viral richness is mainly explained by species richness (suggesting a neutral explanation for the distribution of viral diversity). We accessed mammal order species richness estimates from the International Union for Conservation of Nature and, at each focal year, calculated order-level total viral richness (based on PCR or viral isolation evidence) and virus-related citation counts using only host-virus records up to and including that year. We modelled the relationship between log species richness and viral richness, adjusting for sampling effort (log citations), by fitting generalized linear models with a negative binomial likelihood. Analyses were conducted in R v. 4.0.3 [21].

## 3. Results

Both cumulative discovery curves and fitted GAMs show that viral discovery in mammals is still in an upward growth phase, with little evidence of discovery rates declining towards zero (i.e. viral richness reaching an asymptote) in any group (figure 1; electronic supplementary material, figure S2). This trend is mirrored in virus-related publication counts, which are exponentially increasing year-on-year across most mammal orders and covering an increasingly broad species range (electronic supplementary material, figure S3), but remain unevenly distributed across mammal groups (electronic supplementary material, figure S5). There is evidence for general upticks in discovery rates at two main historical junctures (figure 1), first during the 1960s when technological improvements—including density gradient centrifugation for viral isolation, and establishment of the first human diploid fibroblast cell lines and the now-ubiquitous African green monkey kidney Vero cell line—facilitated industrial-scale production of viruses for research or vaccines [22]. Discovery rates again increased sharply throughout the 2000s, coinciding with improvements in molecular detection techniques and next-generation sequencing, as well as growing funding for viral surveillance in wildlife following the 2002 SARS-CoV epidemic (an uptick in effort that was strongly focused on bats; electronic supplementary material, figure S3). The overall picture is the same at the species level, with the mean cumulative viral richness across all wild species still increasing exponentially (electronic supplementary material, figure S4) and little evidence of discovery rates declining within even highly sampled species (many of which are domestic; electronic supplementary material, figure S6). These trends are very similar when using several more conservative definitions of viral richness (viral genera, ICTV-ratified viruses, or stricter detection criteria excluding serologic detection; electronic supplementary material, figure S7). We also find no evidence that viral richness is becoming more weakly correlated to publication counts over time, as would be expected if viral diversity was reaching an asymptote in well-sampled groups (electronic supplementary material, figure S8).

**Figure 2.**
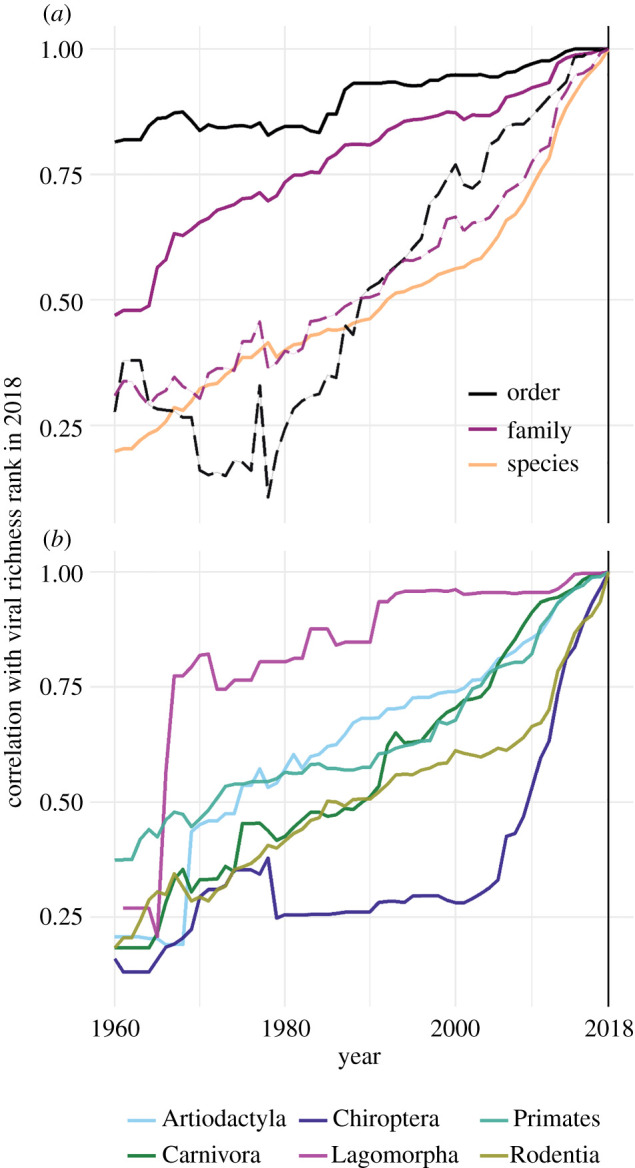
Estimates of relative viral richness across wild mammal taxa are unstable over time. Curves show the rank correlation coefficient (Spearman's *ρ*) between viral richness in 2018 (vertical line) and at annual intervals to 1960. Top panel shows curves for all wild mammals, comparing viral richness at the species level (*n* = 1246), and both total viral richness (solid lines) and mean species-level viral richness (dashed lines) within higher taxonomic groupings (order, *n* = 21; family, *n* = 108) (*a*). Bottom panel shows separate curves of species-level viral richness within six mammalian orders ((*b*); Artiodactyla, *n* = 153 species; Carnivora, *n* = 148; Chiroptera, *n* = 307; Lagomorpha, *n* = 17; Primates, *n* = 157; Rodentia, *n* = 350). Curve shape denotes temporal stability or instability of relative viral richness estimates; a sharper incline corresponds to a faster rearrangement of ranked viral richness (i.e. greater instability) in response to discovery effort.

A consequence of this accelerating discovery trend is that inference of relative viral richness across species and higher taxonomic levels has been unstable over the last 60 years (figure 2). Across all mammals, there is a consistent, gradual temporal decay in rank correlation between present-day and historical estimates of total viral richness, with species-level curves declining much more steeply than those at higher taxonomic levels (dropping to *ρ* = 0.48 by 1991; figure 2*a*). Rankings of mean species-level viral richness at order and family levels (arguably a more relevant metric when considering species contributions to community pathogen maintenance and transmission) are substantially more effort-sensitive than total viral richness, showing much steeper declines (figure 2*a*). Within well-sampled mammal orders there is substantial variation in the historical stability of species-level relative viral richness estimates, and results before 1970 become unstable owing to data sparsity in several orders (figure 2*b*). Notably, within Chiroptera, there has been an extremely rapid reordering of species-level viral richness estimates since 2000 (declining to *ρ* = 0.59 by 2010 and to *ρ* = 0.28 by 2001), probably owing to the ongoing intensification of research effort (electronic supplementary material, figure S3) and viral discovery (figure 1) that followed the emergence of SARS-CoV [23]. Our results show that such rapid changes in host-virus knowledge can impact inference: a positive relationship between order-level species richness and viral richness is only clearly detectable in data from 2010 onwards (electronic supplementary material, figure S9).

## 4. Discussion

Our results suggest that for most mammal species, viral diversity metrics are still shifting and largely reflect historical sampling bias. Given that even well-studied species do not have fully characterized viromes, these estimates are likely to continue shifting in coming years. Inference made on them, however, might become canonical in the literature—and embed false narratives about viral ecology—if these analyses are not repeated as the virome becomes better described. The situation might be improved by massively coordinated projects aiming to accelerate viral discovery [11,24], provided sampling strategies are designed to be taxonomically and geographically representative. However, the rapid recharacterization of the bat virome that has occurred since the first SARS epidemic highlights a significant risk: if sampling strategies are primarily motivated by either existing (zoonotic) viral diversity estimates or health security concerns linked to specific taxa, such initiatives might only further decouple observed and true underlying viral diversity.

Indeed, the unprecedented upward trend in wildlife virus discovery effort since 2000 has been unevenly distributed taxonomically and geographically, with rodents and bats being particularly heavily sampled and showing the highest instability in richness estimates. Ungulates (Artiodactyla and Perissodactyla) are unique among mammals in that reported viral diversity among domestic species exceeds that detected in wildlife (electronic supplementary material, figure S1). Although possibly reflecting the unique ecology of farmed livestock, this more likely reflects a bias towards sampling from livestock, which poses fewer logistical hurdles than sampling from wild ungulates. Further, many viral discovery efforts focus on the detection of targeted viral taxa (e.g. family-level consensus PCR) rather than unbiased approaches that remain cost-prohibitive and analytically challenging. Such evolving detection biases—including efforts to identify bat betacoronaviruses following the emergence of SARS-CoV-2—could, for example, continue to reinforce the perception of certain host taxa as unusually virus-diverse despite inconclusive evidence [13]. Such biases have consequences for the stability of ecological inference: our heuristic analysis demonstrates that the recently reported positive relationship between species richness and viral richness at the order-level [13] only becomes detectable in post-2010 data, which is especially notable given that estimates of relative order-level viral diversity have been more stable than species-level metrics (figure 2). It is therefore concerning that comparative studies of correlates and geographical patterns of host-virus relationships conducted in the mid-2000s might feasibly have drawn quite different conclusions than similar studies conducted now or in the future.

These problems are not necessarily surprising to virologists, who have historically been more hesitant about inference from these limited samples than ecologists, and have encouraged particular caution with respect to inference about human health risks [9]. Multiple studies have found that correcting for undersampling undermines widespread assumptions about zoonotic risk [13,14], and we suggest that future studies should similarly attempt to reject the null hypothesis that downstream patterns of zoonotic risk are a neutral consequence of total observed viral diversity. Given that present-day data are a tiny subset of the latent ‘true’ host-virus network, there will also be value in employing network- or measurement error-based methods that explicitly account for observation biases in analyses [25]. Overall, because current patterns of host-level viral richness represent an unstable and biased snapshot of the mammal virome, we suggest that inference from host-virus association data needs to be carefully qualified and may not by itself be a comprehensive foundation for setting future agendas on viral zoonosis research or One Health policy.

## Data accessibility

Data and code to reproduce the results of this article are archived through Zenodo (https://dx.doi.org/10.5281/zenodo.5720280), with complete documentation provided in the repository Readme file.
